# Supplementary material for: The ctpF Gene Encoding a Calcium P-Type ATPase of the Plasma Membrane Contributes to Full Virulence of Mycobacterium tuberculosis
Source: Int J Mol Sci. 2022 May 27;23(11):6015. doi: 10.3390/ijms23116015 (PMC9180918; doi:10.3390/ijms23116015)

Table S1. Bacterial strains, plasmids and primers used in this study

Strains	Relevant features	Reference
Mycobacterium tuberculosis		
H37Ra	Slow-growing virulent strain, Amp^R, Chx^R, Cb^R	ATCC 25177
H37Rv	Slow-growing attenuated strain, Amp^R, Chx^R, Cb^R	ATCC 25618
H37Rv:pJV53	Recombineering strain (with pJV53), Amp^R, Chx^R, Cb^R, Km^R	This study
H37RvΔ*ctpF*	Δ*ctpF*, gene replaced by a Hyg^R cassette	This study
**Plasmids**	**Relevant features**	**Reference**
pJV53	Derivative of pLAM12 with Che9c 60–61 genes under control of the acetamidase promoter	Gift from Unizar
pYUB854	Hyg^R cassette is flanked by the γδ-res sites and by two MCSs for directional cloning of the homologous recombination substrates	Gift from Unizar
pLNA22	607 bp upstream and 520 bp downstream of *Mtb Rv1997* (*ctpF*) in pYUB854	This study

Primer	Sequence (5'-3')
F-RT Dir	CAGTGATCTTCGGTGTGGTG
F-RT Rev	TGACTCGTTCACGCTCAATC
16SrRNAdir	GAGATAGGCGTTCCCTTGTG
16SrRNArev	CTGGACATAAGGGGCATGAT
RTctpEdir	ACAACGAGCGGGCCTATCCG
RTctpErev	GCCTGTTCCTGCTCCTGCCA
RTctpHdir	TTGCTGCCCGCAATCCTGGA
RTctpHrev	GGCGAGGTCCCGGTGATAGC
A-RT-Dir	GACCACCTCGACGTTGTACC
A-RT-Rev	CAAGCTGTTTGAGACCACGA
I-RT-Dir	CTGTCCTACGAACCGGTGAT
I-RT-Rev	AGTAGCGCGTCGATATTGCT
pJV53dir	GTCAGTCACCAACCCTCCAC
pJV53rev	GAATCCTGCTTGGTGACAGC
ctpF_interno_dir	CTATGCACCCGACGTCCT
cpF_interno_rev	GAACCTGGTATCACGTTTTCG
Comp_Up-ctpF	TCGTCGAACACTCGTACCTG

Comp_Down_ctpF	CGTCCGCAACCTAGTTGAAT
primerpYUB854	GTGGCTCCCTCACTTTCTGG
Hyg_dir_out	ACTTCGAGGTGTTCGAGGAG
Bdir2013	TTTTTCTAGATATCGGGGTGTGGGTGC
Brev2013	TTTTTCATGATACCACCAGCACGATCCAG
Adir2013	TTTTCTCGAGCGGATGGCAAGACC
Arev2013	TTTTGCTAGCGCGCGTTACCACC

Figure S1. Construction of the *Mtb*H37Rv *ctpF* mutant.

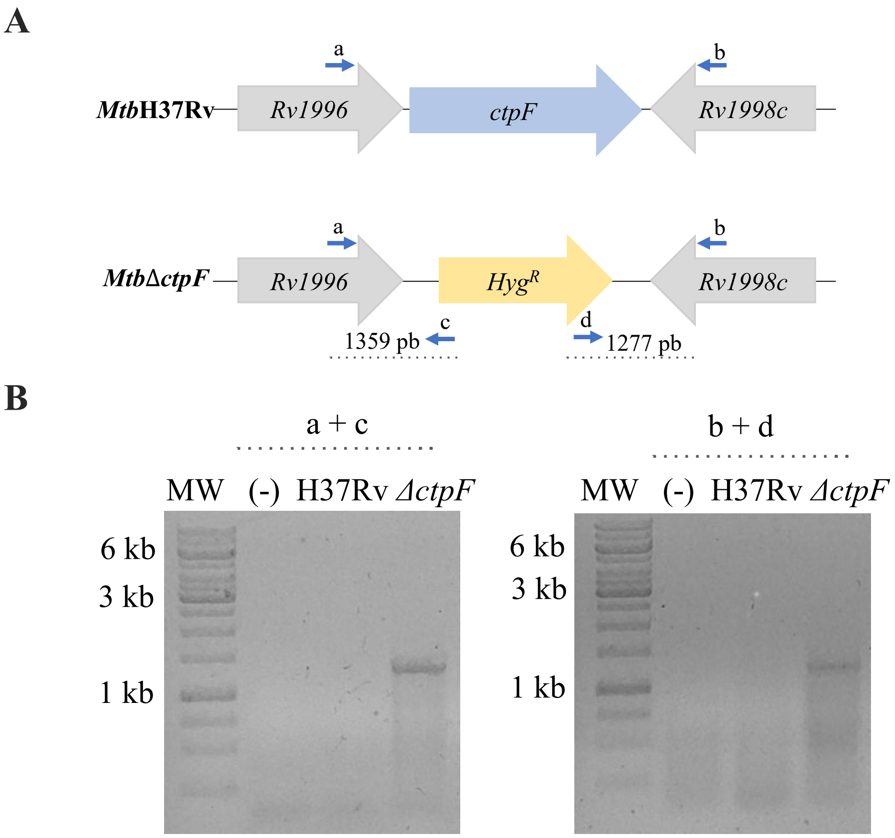

Supplement: Supplementary file 1 [file ijms-23-06015-s001.zip › ijms-1728696-supplementary.pdf]
